# Deciphering Neutral Ceramidase-Dependent Mechanism of Response to Aromatic Fungicides Stress in *Neurospora crassa*

**DOI:** 10.3390/jof12050340

**Published:** 2026-05-06

**Authors:** Pengxu Chen, Yingying Chen, Lize Wang, Ziyi Lan, Xin Zheng, Luoyuan Wang, Xi Gan, Sijia Zhang, Yanxia Zhao

**Affiliations:** School of Life Sciences, Jiangsu Normal University, Xuzhou 221116, China; cpx1469085@outlook.com (P.C.); 2020241812@jsnu.edu.cn (Y.C.); 2020251957@jsnu.edu.cn (L.W.); 3020225257@jsnu.edu.cn (Z.L.); 3020243413@jsnu.edu.cn (X.Z.); 3020243542@jsnu.edu.cn (L.W.); 3020245506@jsnu.edu.cn (X.G.); 3020243282@jsnu.edu.cn (S.Z.)

**Keywords:** filamentous fungi, cellular components, carotenoid, fatty acid, cell wall, cell membrane

## Abstract

Ceramidases hydrolyze ceramides to fatty acids and sphingolipids, but their role in fungal response to stress remains unclear. We investigated the function of neutral ceramidase (nCDase) response to aromatic fungicide (carvacrol, cuminaldehyde, and isoniazid) stress in *Neurospora crassa*. Comparative analysis of the wild-type strain, Δ*nc* and OE*nc* showed that nCDase enhanced fungicide resistance through multiple mechanisms. nCDase improved β-1,3-glucan synthesis (30% increase), decreased membrane permeability, elevated superoxide dismutase and catalase activities, and promoted carotenoid accumulation (50%), which collectively improved stress tolerance. Δ*nc* exhibited disruption of cellular integrity, altered fatty acid profiles (elevated oleic acid, reduced total fatty acids), and increased fungicide sensitivity. Collectively, these findings established that nCDase as a key regulator of cell wall dynamics, lipid homeostasis, and antioxidant defense, thereby facilitating fungal adaptation to abiotic stress. This study identified the role of nCDase in the response to aromatic fungicide stress and laid foundation for inhibiting pathogenic fungi in agricultural production and food preservation.

## 1. Introduction

Sphingolipids are essential structural components of eukaryotic membranes and are key regulators of diverse cellular processes, including programmed cell death, signal transduction, vesicular trafficking, autophagy, alternative splicing, and stress responses [1]. Among the metabolites of sphingolipid, ceramide occupies a central position. It not only serves as a structural component of cell membranes but also as a bioactive signaling molecule that can modulates cell growth, differentiation, and apoptosis [1,2,3]. The metabolic homeostasis of ceramide and its derivatives is tightly controlled by ceramidases (CDases), which hydrolyze ceramide into fatty acids and sphingosine [4]. Based on their optimal pH and subcellular localization, CDases are classified into acid, neutral, and alkaline isoforms, each playing distinct roles in sphingolipid homeostasis [4]. Acid CDases play a role in lysosomal ceramide degradation [5,6], whereas neutral and alkaline CDases are considered to regulate ceramide and sphingosine levels in extracellular and intracellular spaces, respectively, and thus influence cellular responses to environmental stresses [4]. In mammalian cells, sphingosine is not synthesized de novo but is produced through the ceramide hydrolysis by CDase [7]. CDase activity may be a key step in determining the levels of ceramide and sphingosine/sphingosine-1 phosphate, thus playing a crucial role in regulating cell survival or death in response to external stimuli [1,8].

In fungi, sphingolipid metabolism is critical for stress adaptation and membrane integrity. Studies on *Saccharomyces cerevisiae* demonstrated that ceramide and its derivatives regulate cell cycle arrest, thermotolerance, and apoptosis [9,10]. Research on *Sclerotinia sclerotiorum* revealed that the exposure to antifungal compounds (bis-pyrazole carboxamide derivatives) upregulate nCDase activity, leading to ceramide accumulation, oxidative membrane damage, and accelerated cell death [11]. Similarly, a neutral ceramidase (nCDase) orthologue was identified to be involved in fungal pH and temperature responses in *Aspergillus oryzae* [12]. These findings highlight the pivotal role of nCDase in mediating fungal responses to stress. However, despite the advances, the function of nCDase in filamentous fungi remains unknown.

Plant-derived compounds such as carvacrol, cinnamaldehyde, and isoniazid have emerged as promising antifungal agents due to their ability to disrupt membrane integrity and induce oxidative stress [13,14]. Carvacrol is a monoterpenoid phenol with broad-spectrum activity against fungal pathogens, including *Fusarium* and *Aspergillus* [13,14,15]. Notably, carvacrol enhances the efficacy of traditional antifungal agents, suggesting potential synergistic application [16]. Cuminaldehyde demonstrated antimicrobial properties against *Penicillium italicum* [17]. Isoniazid was originally developed as an antitubercular agent, but research indicates its potential in controlling wheat crown rot caused by *Fusarium pseudograminearum* [18]. Despite the apparent efficacy of these compounds, the molecular mechanism of their antifungal action remains to be explored.

To address these questions, we used the fungus *Neurospora crassa* as a model to decipher the mechanism by which fungi depend on nCDase in response to fungicide stress, focusing on three different antifungal agents: carvacrol, cuminaldehyde, and isoniazid. This work provides novel insights into the role of nCDase (NCU04721) in fungal stress adaptation and informs the development of novel antifungal strategies.

## 2. Materials and Methods

### 2.1. Strains and Growth Conditions

*N. crassa* strains were cultured in solid minimal medium (50× Vogel’s 20 mL/L, Sucrose 30 g/L, and Agar 15 g/L) or medium containing quinic acid (50× Vogel’s 20 mL/L, Glucose 1 g/L, Arginine 1.7 g/L, Sorbinose 2 g/L, Quinic acid 0.01 mol/L, and Agar 15 g/L) [19]. Carvacrol (CAS: 499-75-2), cuminaldehyde (CAS: 122-03-2), and isoniazid (CAS: 54-85-3) were acquired from Macklin (Shanghai, China). Each compound was dissolved in 0.1% Tween 80 solution and the mixture of the three compounds was prepared by weighing out carvacrol, cuminaldehyde and isoniazid in a ratio of 1:10:50 (the proportions of the mixture were determined based on the strongest inhibition in the pre-experiment). Appropriate volumes of these solutions were added to the culture medium as required.

### 2.2. Generation of nc Mutants

*nc* deletion mutant (Δ*nc*) was obtained from the Fungal Genetics Stock Center (FGSC). For the overexpression mutants (OE*nc*), nCDase open reading frame was cloned into pqa-5Myc-6His which contained quinic acid-induced qa-2 promoter [20,21]. The plasmid was then introduced into the Δ*nc*/Δ*his*-3 recipient strain to generate OE*nc*. OE*nc* was cultured on the medium with quinic acid.

### 2.3. Growth and Development Under Fungicides Stress

To assess the growth and development of the strains under fungicide stress, each strain was inoculated onto solid medium with different fungicides. Based on the preliminary inhibition assays, the concentration ranges were set as follows: 50 to 300 μg mL^−1^ for carvacrol, 100 to 300 μg mL^−1^ for cuminaldehyde, 100 to 600 μg mL^−1^ for isoniazid, and 50 to 150 μg mL^−1^ for the mixture of these fungicides. The antifungal experiments were carried out on solid minimal medium at 30 °C for 7 d. The *N. crassa* FGSC4200 strain was used as the wild-type strain. The fungicide-free group was used as the control. All antifungal experiments were performed in three plates from the same culture.

### 2.4. Observation of Mycelial Morphological Structure

The morphological structure of mycelia was examined using lactophenol cotton blue stain [22]. Briefly, *N. crassa* were inoculated at the center of plates with different fungicides. And sterilized coverslips were inserted at 45°, about 2 cm from the inoculation point. After 24 h of incubation at 30 °C, the coverslips with mycelia were collected. For staining, 3 drops of lactophenol cotton blue stain were placed on the slide and a coverslip was placed over the stain to form a wet mount. The morphology was then observed under a microscope.

The coverslips covered with mycelium were collected, and 3 drops of 2.5% (*v*/*v*) glutaraldehyde solution were added to cover the mycelium, and fixed at 4 °C for 4 h. Then rinse the coverslips 3 times with ddH_2_O to remove the residual glutaraldehyde, and then place in a cool place to dry, spray the coverslips with gold, and observe the ultrastructure of mycelium with a scanning electron microscope [22].

### 2.5. Quantification of Chitin Content

Chitin content was determined through enzymatic hydrolysis followed by colorimetric analysis. Chitinase hydrolyzes chitin into N-acetylglucosamine. It reacts with p-dimethylaminobenzaldehyde to form red chromophore with maximum absorbance at 530 nm. The cultures from different strains under different stress were dried at 65 °C for 12 h and treated with concentrated hydrochloric acid at 25 °C for 24 h. The acid solution was diluted to 8.5 mol L^−1^ with ddH_2_O and digested in boiling water bath for 2 h. After cooling, the pH was adjusted to neutral by NaOH solution, and the mixture was filtered through filter paper. For the colorimetric assay, the processed sample was mixed with acetylacetonate reagent (take 1.50 mL of acetylacetone, dissolve it in 50 mL of 1.25 mol/L Na_2_CO_3_ solution and mix well. Prepare it just before use) and incubated at 90 °C for 1 h. Following cooling to room temperature, anhydrous ethanol was added and then left to stand for 1 h at room temperature. The absorbance at 530 nm was measured using a Multi-mode Microplate Reader (Synergy H4, Bio Tek, Winooski, VT, USA). A standard curve was prepared with N-acetylglucosamine to calculate the chitin content [23]. All measurements were done in three technical measurements.

### 2.6. Trehalose Quantification Assay

To determine trehalose content, 7-day cultures were collected, resuspended in ddH_2_O, and incubated at 95 °C for 30 min to extract intracellular trehalose. The supernatant was mixed with an equal volume of 0.2 mol L^−1^ sodium citrate and divided into two aliquots: one was treated with trehalase, while the other was not (as the negative control). After incubation at 37 °C for 8 h, the glucose released from trehalose hydrolysis was quantified using a Glucose Assay Kit Item No. A149-1-1 (Nanjing JianCheng Bioengineering Institute, Nanjing, China) in three technical measurements.

### 2.7. β-Glucan Quantification Assay

The β-glucan content was determined by aniline blue fluorescence assay. In brief, the cultures were ground in liquid nitrogen and then extracted with water at 100 °C for 2 h. The extract was filtered through 200-mesh filter screen. The extraction process was repeated 3 times to ensure complete recovery. The combined filtrates were centrifuged at 3000× *g* for 15 min and concentrated to 1 mL. For assay, 300 μL of the extract was mixed with 30 μL of 6 mol L^−1^ KOH and incubated for 30 min at 80 °C, followed by immediate cooling in an ice bath. Subsequently, the temporary prepared aniline blue solution (aqueous aniline blue: HCl: glycine buffer vol. ration of 40:21:59) was added, and the solution was incubated at 50 °C for 30 min. Fluorescence intensity was measured using a Multi-mode Microplate Reader with excitation at 398 nm and emission at 502 nm [24]. All measurements were performed in three technical measurements.

### 2.8. Extraction and Quantification of Ergosterol

Ergosterol was extracted using ultrasonic crushing method with methanol as extraction solvent. Briefly, the cultures were pulverized with liquid nitrogen. The power was subjected to methanol with ultrasonication (400 W) (JY92-IIN, Ningbo scientz biotechnology Co., Ltd., Ningbo, China) for 80 min. The extract was centrifugated at 2000× *g* for 10 min and the supernatant was collected. The extraction process was repeated 3 times. The combined supernatants were concentrated to 2 mL and filtered through 0.22 μm filter membrane. Ergosterol content was determined by high-performance liquid chromatography (HPLC) (Waters e2695, Waters Corporation, Milford, MA, USA) using Atlantis dC18 column (3 μm, 2.1 mm × 150 mm) at 30 °C. The mobile phase was 100% methanol at a flow of 0.3 mL min^−1^. Detection was carried out at 283 nm. Ergosterol content was quantified by peak area normalization method using ergosterol standard curve [25].

### 2.9. Determination of Cell Membrane Integrity

Cell membrane permeability was evaluated by quantifying the release of intracellular components. Spore suspensions of different strains were treated with carvacrol, cuminaldehyde, isoniazid, or the mixture, and incubated at 30 °C for 1 h with continuous shaking (150 rpm). After incubation, the samples were centrifuged at 1000× *g* for 5 min. The supernatant was collected and the absorbance at 260 nm was measured to assess nucleic acid release [26]. Three replicates were performed.

The integrity of cytoplasmic membrane was evaluated using propidium iodide (PI). PI is a fluorescent dye that cannot penetrate intact cell membranes, but can enter mid–late apoptotic and dead cells to stain double-stranded DNA red, and is commonly used to detect cell death or changes in membrane permeability. Spores were collected and washed twice with phosphate-buffered saline (PBS) and resuspended in 500 μL PBS. Subsequently, 5 μL of PI staining solution was added to the suspension, mixed, and incubated in the dark on ice for 15 min. Fluorescence intensity was measured using a Multi-mode Microplate Reader with excitation at 535 nm and emission at 615 nm. Membrane integrity was quantified based on the relative fluorescence intensity of PI, with higher values indicating greater membrane damage [27]. Three independent replicates were performed.

### 2.10. Crude Protein Extraction

Fungicide-treated and untreated *N. crassa* cultures were ground in liquid nitrogen, vortexed after the addition of Hepes extraction buffer containing 1 mmol L^−1^ PMSF, and ice-bathed for 5 min. The homogenate was centrifuged at 8000× *g* for 10 min at 4 °C. Subsequently, the supernatant was transferred to microcentrifuge tubes. The extraction was repeated 3 times and the supernatants were combined to form crude protein solution. Protein concentration was determined by the Coomassie Brilliant Blue Method using Calf Serum as a standard [28].

### 2.11. Measurement of Catalase Activity

Catalase (CAT) activity was determined by measuring its ability to decompose H_2_O_2_. The enzymatic reaction was terminated by adding ammonium molybdate, which reacts with residual H_2_O_2_ to form a stable yellow-colored complex, and the amount of change can be measured at 405 nm, which can be used to calculate the activity of CAT. CAT activity was detected by referring to the CAT assay kit (Item No. A007-1-1, Nanjing JianCheng Bioengineering Institute, Nanjing, China).

### 2.12. Determination of Total Superoxide Dismutase Activity

Total superoxide dismutase (T-SOD) activity was determined by the xanthine oxidase method. T-SOD activity was carried out according to the instructions of T-SOD Assay Kit (Item No. A001-1) from Nanjing JianCheng Bioengineering Institute (Nanjing, China) and OD_550_ was read by a Multi-mode Microplate Reader.

### 2.13. Determination of Malondialdehyde Content

Malondialdehyde (MDA) is a product of lipid peroxidation, which can react with thiobarbituric acid to form a red product with maximum absorption at 532 nm. Referring to the instructions of malondialdehyde test kit Item No. A003-1 (Nanjing JianCheng Bioengineering Institute, Nanjing, China) to detect MDA content in *N. crassa*.

### 2.14. Identification of Carotenoid Composition

The cultures from fungicides stress were ground with liquid nitrogen. Carotenoids were extracted 3 times by ultrasonication using acetone as the extractant. The content and composition of carotenoids were determined by HPLC [21,29].

### 2.15. Fatty Acids Profile Analysis

The effects of nCDase and fungicides on fatty acid production were assessed by gas chromatography (GC) (Agilent 8860, Agilent Technologies, Inc., Santa Clara, CA, USA). Briefly, fatty acids were extracted with petroleum ether using flash extractor. The concentrated sample was placed in a 10 mL tube, and 2 mol L^−1^ sodium hydroxide methanol solution was added, mixed and sealed, and then placed in a water bath at 70 °C and shaken for 25 min. After cooling, n-hexane was added, and the n-hexane phase was filtered through 0.22 μm filter membrane. Fatty acid content and composition was performed using GC with an Agilent Eclipse XDB C_8_ column. Fatty acids were analyzed quantitatively and qualitatively based on peak areas of standard [30].

### 2.16. Statistical Analysis

Statistical analysis was performed using SPSS 20.0. All data are expressed as mean ± standard deviation (SD). Comparisons between the treatment group and the control group were performed using Student’s *t*-test. *p*-value < 0.05 was considered as significantly different.

## 3. Results

### 3.1. Phenotypes of nCDase Mutants

**Figure 1:**
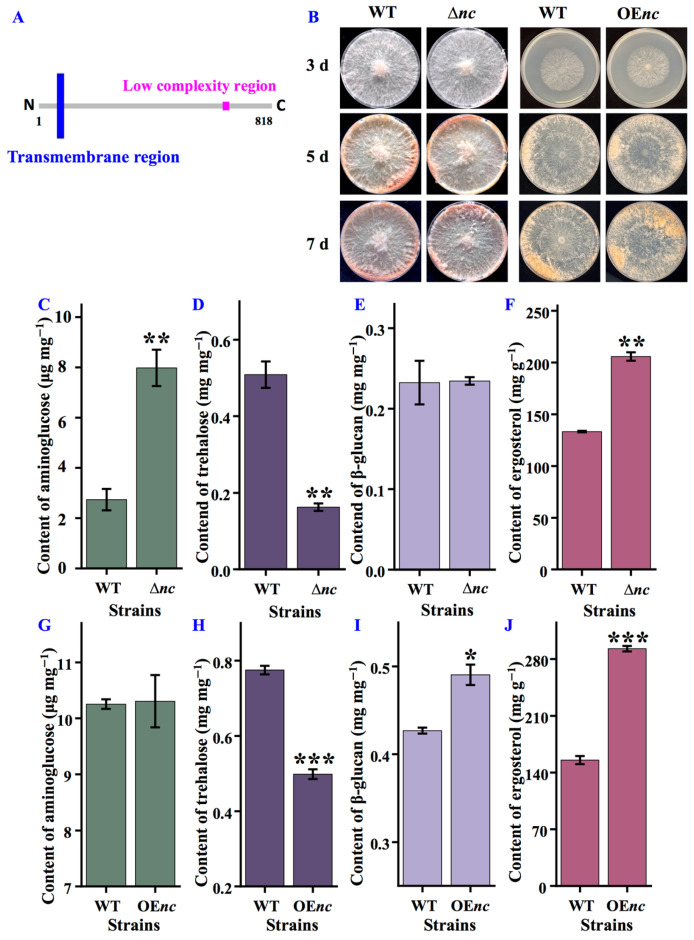
nCDase is important for cell wall and cell membrane formation in *N. crassa*. (**A**) Domain structure of nCDase by SMART database (http://smart.embl-Heidelberg.de (accessed on 1 December 2023)) and Protein Blast (http://blast.ncbi.nlm.nih.gov (accessed on 1 December 2023)). (**B**) Growth phenotypes of wild-type, Δ*nc*, and OE*nc* strains grown on solid media at 37 °C for 7 days. (**C**) The quantified aminoglucose levels in wild-type and Δ*nc* strains. (**D**) The quantified trehalose levels in wild-type and Δ*nc* strains. (**E**) The quantified β-glucan levels in wild-type and Δ*nc* strains. (**F**) The quantified ergosterol levels in wild-type and Δ*nc* strains. (**G**) The quantified aminoglucose levels in wild-type and OE*nc* strains. (**H**) The quantified trehalose levels in wild-type and OE*nc* strains. (**I**) The quantified β-glucan levels in wild-type and OE*nc* strains. (**J**) The quantified ergosterol levels in wild-type and OE*nc* strains. WT, the wild-type strain. Δ*nc* was cultured in on solid minimal media, while OE*nc* was grown in medium with quinic acid; the wild-type strain was used as a control under both culture conditions. Values are means ± SD (*n* indicates triplicates), and error bars represent SD. Asterisks indicate comparisons between *nc* mutant and wild-type strain. * *p* < 0.05, ** *p* < 0.01, and *** *p* < 0.001.

Genetic sequence analysis revealed that nCDase (GeneBank: EAA31120.3) consists of 818 amino acids and contains one transmembrane domain (Figure 1A). To investigate the effects of nCDase on *N. crassa* development, Δ*nc* was inoculated on the solid minimal medium and OE*nc* on the medium containing quinic acid, respectively. As shown in Figure 1B, there was no significant difference in the growth phenotypes of Δ*nc* and OE*nc* compared to the wild-type strain of *N. crassa*. This indicates that nCDase has no significant effect on the growth rate and morphology of *N. crassa*.

### 3.2. nCDase Is Essential for Cell Wall and Cell Membrane

Analysis of the composition of the cell wall and cell membrane in different strains showed significant changes. Compared with the wild-type strain, Δ*nc* exhibited increased levels of chitin (3.0-fold) (Figure 1C) and ergosterol (1.5-fold) (Figure 1F). Conversely, Δ*nc* showed a decrease in trehalose content (3.0-fold) (Figure 1D). Meanwhile, OE*nc* demonstrated no significant difference in chitin accumulation compared to the wild-type strain (Figure 1G). However, OE*nc* displayed elevated levels of β-glucan (1.1-fold) (Figure 1I) and ergosterol (1.9-fold) (Figure 1J), accompanied by 1.5-fold decrease in trehalose content (Figure 1H).

### 3.3. nCDase Regulates the Production of Carotenoids

**Figure 2:**
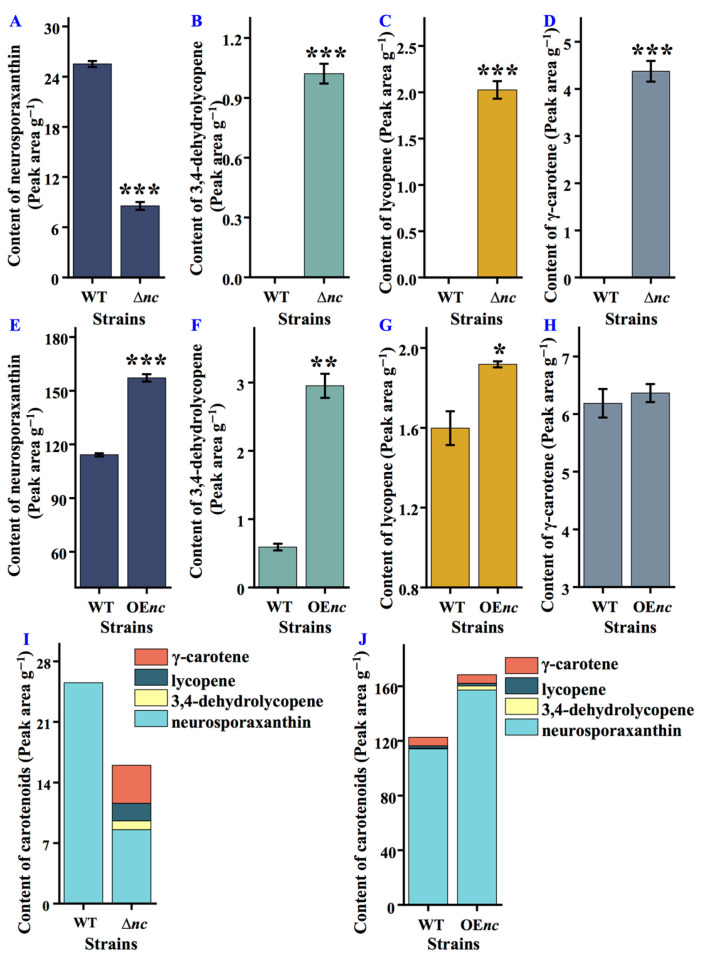
nCDase contributes to carotenoids biosynthesis in *N. crassa*. (**A**) Content of neurosporaxanthin in wild-type and Δ*nc* strains. (**B**) Content of 3,4-dehydrolycopene in wild-type and Δ*nc* strains. (**C**) Content of lycopene in wild-type and Δ*nc* strains. (**D**) Content of γ-carotene in wild-type and Δ*nc* strains. (**E**) Content of neurosporaxanthin in wild-type and OE*nc* strains. (**F**) Content of 3,4-dehydrolycopene in wild-type and OE*nc* strains. (**G**) Content of lycopene in wild-type and OE*nc* strains. (**H**) Content of γ-carotene in wild-type and OE*nc* strains. (**I**) Content of carotenoids in wild-type and Δ*nc* strains. (**J**) Content of carotenoids in wild-type and OE*nc* strains. WT, the wild-type strain. Δ*nc* was cultured in on solid minimal media, while OE*nc* was grown in medium with quinic acid; the wild-type strain was used as a control under both culture conditions. Values are means ± SD (*n* indicates triplicates), and error bars represent SD. Asterisks indicate comparisons between *nc* mutant and wild-type strain. * *p* < 0.05, ** *p* < 0.01, and *** *p* < 0.001.

HPLC analysis revealed changes in carotenoid profiles between *nc* mutants and the wild-type strain of *N. crassa*. Compared to the wild-type strain, the total carotenoids content in Δ*nc* decreased (Figure 2I), with reduced synthesis in neurosporaxanthin synthesis (Figure 2A), which accounted for over 66.5% of total carotenoids, while 3,4-dehydrolycopene (Figure 2B), lycopene (Figure 2C), and γ-carotene (Figure 2D) increased. In contrast, OE*nc* displayed an overall increase in total carotenoids content, with elevated levels of neurosporaxanthin, 3,4-dehydrolycopene, and lycopene (Figure 2E–G,J). These findings suggest that nCDase plays a regulatory role in carotenoids’ biosynthesis. The absence of *nc* disrupted neurosporaxanthin synthesis, leading to the accumulation of precursors. Conversely, *nc* overexpression enhanced the activity of the carotenoid synthesis pathway and promoted the production of all detected carotenoids, including neurosporaxanthin.

### 3.4. nCDase Affects T-SOD and CAT Activities

**Figure 3:**
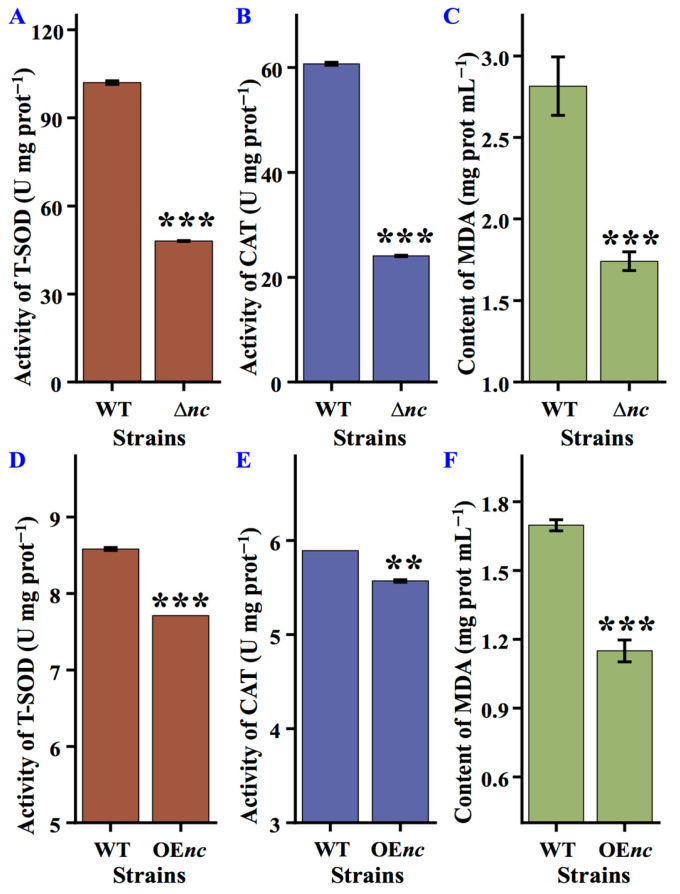
Activity of antioxidant enzyme in wild-type and *nc* mutants of *N. crassa*. (**A**) T-SOD activity in wild-type and Δ*nc* strains. (**B**) CAT activity in wild-type and Δ*nc* strains. (**C**) Content of MDA in wild-type and Δ*nc* strains. (**D**) T-SOD activity in wild-type and OE*nc* strains. (**E**) CAT activity in wild-type and OE*nc* strains. (**F**) Content of MDA in wild-type and OE*nc* strains. WT, the wild-type strain; T-SOD, total superoxide dismutase; CAT, catalase; MDA, malondialdehyde. Δ*nc* was cultured in on solid minimal media, while OE*nc* was grown in medium with quinic acid; the wild-type strain was used as a control under both culture conditions. Values are means ± SD (*n* indicates triplicates), and error bars represent SD. Asterisks indicate comparisons between *nc* mutant and wild-type strain. ** *p* < 0.01, and *** *p* < 0.001.

The enzymatic activities of T-SOD and CAT in wild-type, Δ*nc*, and OE*nc* strains were analyzed using colorimetric methods. Deletion and overexpression of *nc* resulted in reduction of T-SOD (102.04 ± 0.62 U mg prot^−1^ vs. 48.09 ± 0.15 U mg prot^−1^, 8.58 ± 0.02 U mg prot^−1^ vs. 7.71 ± 0.0 U mg prot^−1^) (Figure 3A,D) and CAT activities (60.72 ± 0.29 U mg prot^−1^ vs. 24.08 ± 0.13 U mg prot^−1^, 5.89 ± 0.0 U mg prot^−1^ vs. 5.57 ± 0.01 U mg prot^−1^) (Figure 3B,E), accompanied by decreased MDA levels (2.81 ± 0.17 mg prot mL^−1^ vs. 1.74 ± 0.06 mg prot mL^−1^, 1.70 ± 0.02 mg prot mL^−1^ vs. 1.15 ± 0.05 mg prot mL^−1^) (Figure 3C,F). These suggest that *nc* not only regulates the activities of T-SOD and CAT but also influences the synthesis or activity of other antioxidant components to reduce lipid peroxidation.

### 3.5. Determination of Inhibitory Concentrations of Fungicides

**Figure 4:**
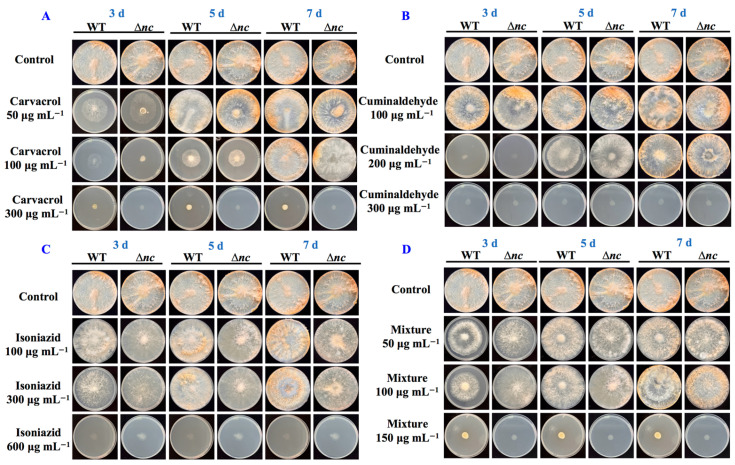
The impact of aromatic fungicides on phenotype of wild-type and Δ*nc* strains. (**A**) Phenotype of wild-type and Δ*nc* strains upon carvacrol stress. (**B**) Phenotype of wild-type and Δ*nc* strains upon cuminaldehyde stress. (**C**) Phenotype of wild-type and Δ*nc* strains upon isoniazid stress. (**D**) Phenotype of wild-type and Δ*nc* strains upon the mixture stress. WT, the wild-type strain. The mixture of the three compounds was prepared by weighing out carvacrol, cuminaldehyde and isoniazid in a ratio of 1:10:50. Strains grown on solid minimal media with or without aromatic fungicides stress at 37 °C.

We evaluated the sensitivity of *N. crassa* to carvacrol, cuminaldehyde, isoniazid, and their mixture by concentration gradient experiments. The final concentrations of carvacrol were 50, 100, and 300 μg mL^−1^. The final concentrations of cuminaldehyde were 100, 200, and 300 μg mL^−1^. The final concentrations of isoniazid were 100, 300, and 600 μg mL^−1^. And the final concentrations of carvacrol: cuminaldehyde: isoniazid (1:10:50) were 50, 100, and 150 μg mL^−1^. All experimental groups exhibited concentration dependent inhibition of *N. crassa* growth. 100 μg mL^−1^ carvacrol showed significant growth inhibition within 5 days of incubation, while 300 μg m^L−1^ carvacrol achieved complete inhibition (Figure 4A). An amount of 200 μg mL^−1^ cuminaldehyde inhibited mycelial growth at 3-day incubation, whereas 300 μg mL^−1^ completely inhibited the growth of *N. crassa* (Figure 4B). The inhibitory threshold of isoniazid was higher. It showed no significant inhibitory activity at 300 μg mL^−1^, but completely inhibited the growth of *N. crassa* at 600 μg mL^−1^ (Figure 4C). The mixture stress exhibited a significant synergistic effect, inhibiting the mycelial extension of *N. crassa* at 50 μg mL^−1^ and completely inhibiting its growth at 150 μg mL^−1^ (Figure 4D). Compared to carvacrol, cuminaldehyde, and isoniazid, the mixture significantly reduced the concentration required for complete inhibition, indicating that the three components enhanced the inhibitory efficiency through synergistic effects. Additionally, the wild-type strain demonstrated stronger activity against carvacrol stress and Δ*nc* showed stronger activity against the mixture stress (Figure 4).

### 3.6. Aromatic Fungicide-Induced Morphogenetic Disruption

**Figure 5:**
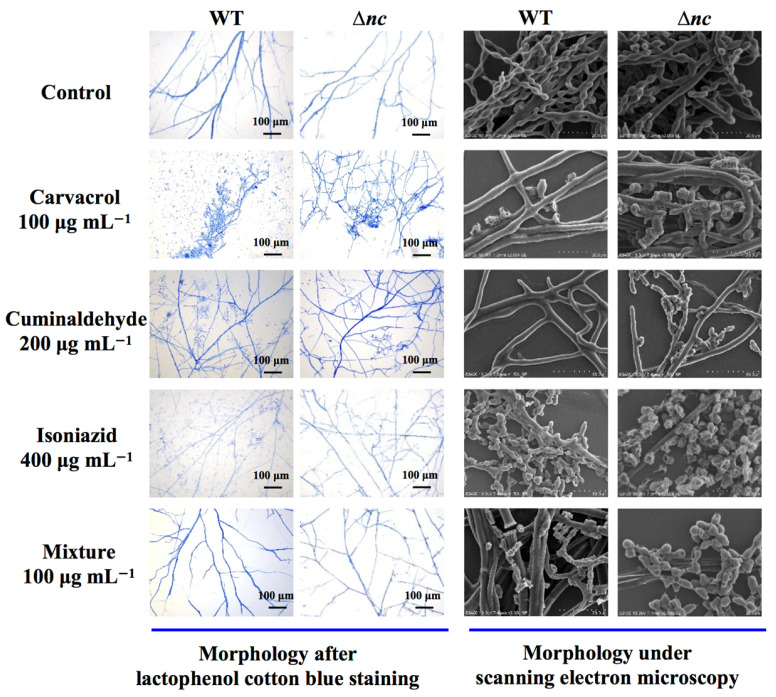
The impact of aromatic fungicides on the morphology of the wild-type and Δ*nc* strains. WT, the wild-type strain. The mixture of the three compounds was prepared by weighing out carvacrol, cuminaldehyde and isoniazid in a ratio of 1:10:50.

Lactophenol cotton blue staining is a classic microbiological technique for observing mycelium, as it selectively binds to the chitin in the cell wall, enhancing microscopic resolution of mycelia. The mycelial morphogenesis of the wild-type and Δ*nc* strains was observed by electron microscopy and scanning electron microscopy. Under normal culture condition, both the wild-type and Δ*nc* exhibited branched mycelia with conidial chains, smooth surfaces, and characteristic contraction-phase morphology with interconnected spores (Figure 5). Exposure of *N. crassa* to aromatic fungicides induced morphological changes. Δ*nc* presented mycelial breaks, crumpling and depression of conidia (Figure 5).

### 3.7. nCDase Is Essential for Integrity of Cell Membrane Under Stress

**Figure 6:**
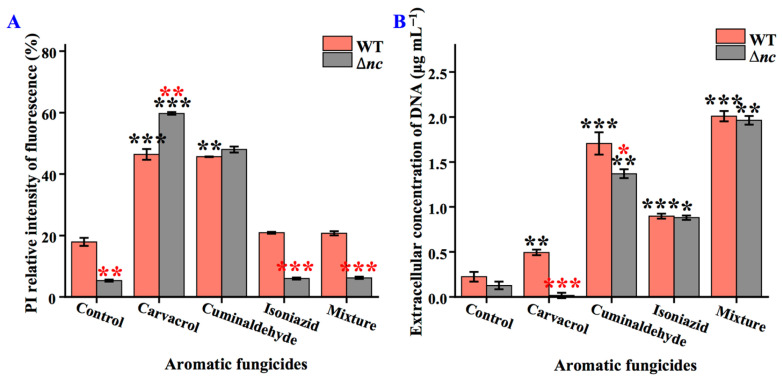
Effects of aromatic fungicides on PI relative fluorescence intensity (**A**) and extracellular DNA concentration (**B**) in the wild-type and Δ*nc* strains. WT, the wild-type strain. The mixture of the three compounds was prepared by weighing out carvacrol, cuminaldehyde and isoniazid in a ratio of 1:10:50. Values are means ± SD (*n* indicates triplicates), and error bars represent SD. Black asterisks indicate comparisons of the same strain under aromatic fungicide treatment and normal culture conditions; red asterisks indicate comparisons of Δ*nc* and wild-type strains under the same culture conditions. * *p* < 0.05, ** *p* < 0.01, and *** *p* < 0.001.

To investigate the effect of nCDase on cell membrane permeability, we measured the relative fluorescence intensity of PI and the extracellular DNA concentration. PI is a membrane non-permeable dye that enters cell through damaged cell membrane and binds to DNA or RNA. This method assesses the integrity of the cell membrane by detecting the fluorescence intensity of the cells after PI staining. As shown in Figure 6, both the wild-type and Δ*nc* strains exhibited low relative fluorescence intensity of PI and extracellular DNA concentration. Treatment with carvacrol (46.40 ± 1.73 and 59.74 ± 0.41) and cuminaldehyde (45.64 ± 0.12 and 47.98 ± 0.96) increased the relative fluorescence intensity of PI in both wild-type and Δ*nc* strains. Upon isoniazid and the mixture treatments, there was no significant change in the relative fluorescence intensity of PI between the wild-type and Δ*nc* (Figure 6A), but the extracellular DNA concentration increased (Figure 6B).

Under normal incubation, isoniazid and the mixture treatments, the relative fluorescence intensity of Δ*nc* was lower than that of the wild-type strain, but there was no significant difference in extracellular DNA concentration (Figure 6). Upon carvacrol treatment, the PI relative fluorescence intensity in Δ*nc* was higher than that in the wild-type strain, but the extracellular DNA concentration was lower than that in the wild-type strain (Figure 6).

### 3.8. Regulation of nCDase on Cell Wall and Cell Membrane Remodeling Under Stress

**Figure 7:**
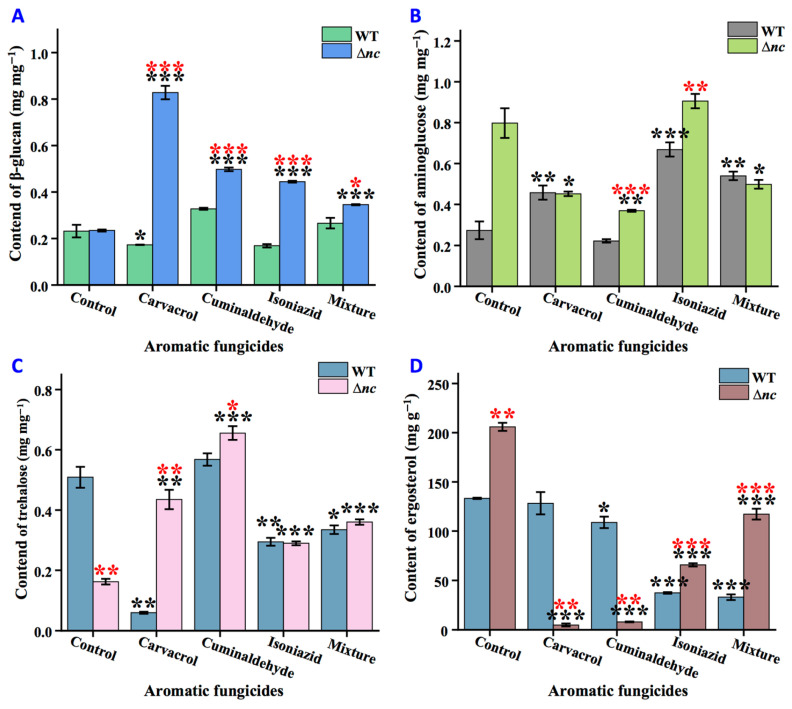
Effects of aromatic fungicides on cell wall and membrane components in wild-type and Δ*nc* strains. (**A**) The content of β-glucan. (**B**) The content of chitin. (**C**) The content of trehalose. (**D**) The content of ergosterol. WT, the wild-type strain. The mixture of the three compounds was prepared by weighing out carvacrol, cuminaldehyde and isoniazid in a ratio of 1:10:50. Values are means ± SD (*n* indicates triplicates), and error bars represent SD. Black asterisks indicate comparisons of the same strain under aromatic fungicide treatment and normal culture conditions; red asterisks indicate comparisons of Δ*nc* and wild-type strains under the same culture conditions. * *p* < 0.05, ** *p* < 0.01, and *** *p* < 0.001.

The synthesis of major components of the cell wall and cell membrane under the different aromatic fungicide stresses was investigated. As shown in Figure 7, under carvacrol treatment, chitin content of the wild-type strain increased by 67.37%, while the trehalose content decreased by 88.25%; in Δ*nc*, β-glucan and trehalose content increased by 253.46% and 167.56%, while ergosterol content decreased. After treatment with cuminaldehyde, there was no significant differences in the content of β-glucan, chitin, and trehalose in the wild-type strain. In Δ*nc*, the contents of trehalose and β-glucan increased by about 3 times and 2 times respectively, while the contents of other components decreased. Under isoniazid treatment, chitin content of the wild-type strain increased by 1.4-fold and ergosterol content decreased by 74%; in Δ*nc*, the content of chitin and trehalose increased, and the β-glucan and ergosterol contents decreased. Under the mixture treatment, the chitin content of the wild-type strain increased, but trehalose and ergosterol contents decreased; Δ*nc* showed a decrease in chitin and ergosterol contents, but an increase in trehalose and β-glucan contents (Figure 7).

Comparison of the wild-type and Δ*nc* under the same culture conditions revealed that, under normal culture conditions, only the trehalose content of Δ*nc* strain was lower than that of the wild-type strain. Upon the treatment of carvacrol, trehalose and β-glucan contents were higher than those of the wild-type strain, while the content of ergosterol was reduced. Upon cuminaldehyde treatment, β-glucan, trehalose, and chitin contents were all higher than those of the wild-type strain. And under the mixture treatment, the content of chitin did not exceed that of the wild-type strain (Figure 7).

### 3.9. nCDase Affects the Composition of Carotenoids and Stress Response

**Figure 8:**
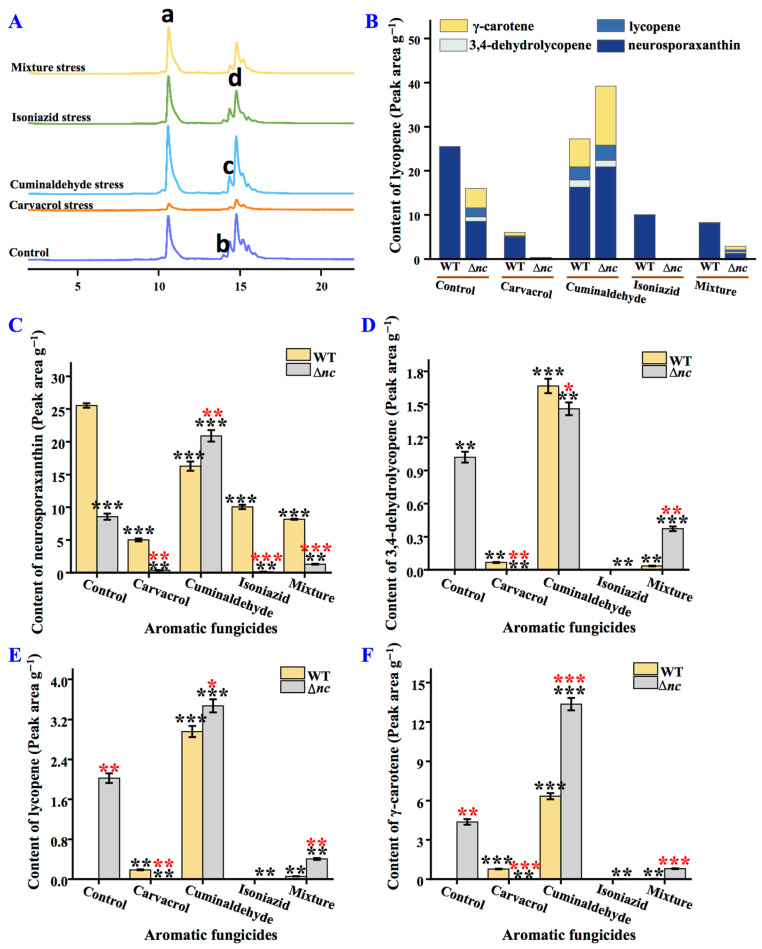
Effects of aromatic fungicides on carotenoids production in wild-type and Δ*nc* strain. (**A**) Chromatogram of carotenoids by HPLC. a, neurosporaxanthin; b, 3,4-dehydrolycopene; c, lycopene; d, γ-carotene. (**B**) The content of total carotenoids. (**C**) The content of neurosporaxanthin. (**D**) The content of 3,4-dehydrolycopene. (**E**) The content of lycopene. (**F**) The content of γ-carotene. WT, the wild-type strain. The mixture of the three compounds was prepared by weighing out carvacrol, cuminaldehyde and isoniazid in a ratio of 1:10:50. Values are means ± SD (*n* indicates triplicates), and error bars represent SD. Black asterisks indicate comparisons of the same strain under aromatic fungicide treatment and normal culture conditions; red asterisks indicate comparisons of Δ*nc* and wild-type strains under the same culture conditions. * *p* < 0.05, ** *p* < 0.01, and *** *p* < 0.001.

The carotenoid content and composition of *N. crassa* undergo changes under the stress of aromatic fungicides. As shown in Figure 8, under the treatment of cuminaldehyde, the total carotenoid content of Δ*nc* increased, but decreased under other treatments. Furthermore, the accumulation of 3,4-dehydrolycopene, lycopene, and γ-carotene improved under cuminaldehyde stress. In contrast, the content of neurosporaxanthin decreased in the wild-type strain under different treatment conditions, and the accumulation of 3,4-dehydrolycopene, lycopene, and γ-carotene increased under the treatment of cuminaldehyde. In addition, comparison of the wild-type and Δ*nc* under the same culture conditions revealed that, except for Δ*nc* which had higher carotenoid content under cuminaldehyde treatment, the wild-type strain had higher carotenoid content under all other conditions (Figure 8).

### 3.10. Effect of Aromatic Fungicides on CAT and T-SOD Activities

**Figure 9:**
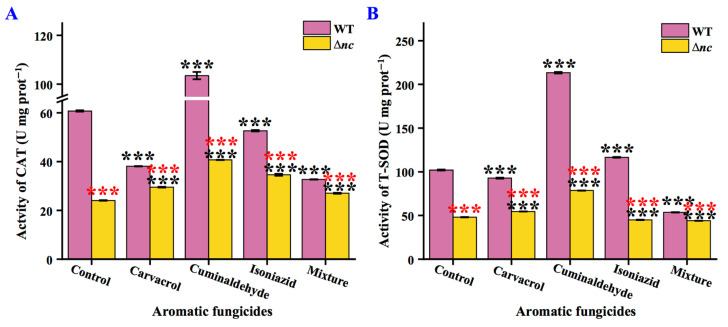
Effects of aromatic fungicides on antioxidant activity in wild-type and Δ*nc* strain. (**A**) The activity of CAT. (**B**) The activity of T-SOD. WT, the wild-type strain; CAT, catalase; T-SOD, total superoxide dismutase. The mixture of the three compounds was prepared by weighing out carvacrol, cuminaldehyde and isoniazid in a ratio of 1:10:50. Values are means ± SD (*n* indicates triplicates), and error bars represent SD. Black asterisks indicate comparisons of the same strain under aromatic fungicide treatment and normal culture conditions; red asterisks indicate comparisons of Δ*nc* and wild-type strains under the same culture conditions. *** *p* < 0.001.

The effect of aromatic fungicides on the activities of antioxidant enzymes in *N. crassa* was studied. CAT and T-SOD activities of the wild-type strain were reduced under carvacrol and mixture treatments, whereas they were elevated by 70.49% and 109.10% under cuminaldehyde treatment. With cuminaldehyde stress, CAT and T-SOD activities in Δ*nc* were increased by 68.93% and 63.52%, respectively, while under other treatments, CAT and T-SOD activities were not significantly increased in Δ*nc* (Figure 9). Additionally, CAT and T-SOD activities in Δ*nc* were lower than those of wild-type strain under the same culture conditions (Figure 9).

### 3.11. The Absence of nc Affects the Composition of Fatty Acids

**Figure 10:**
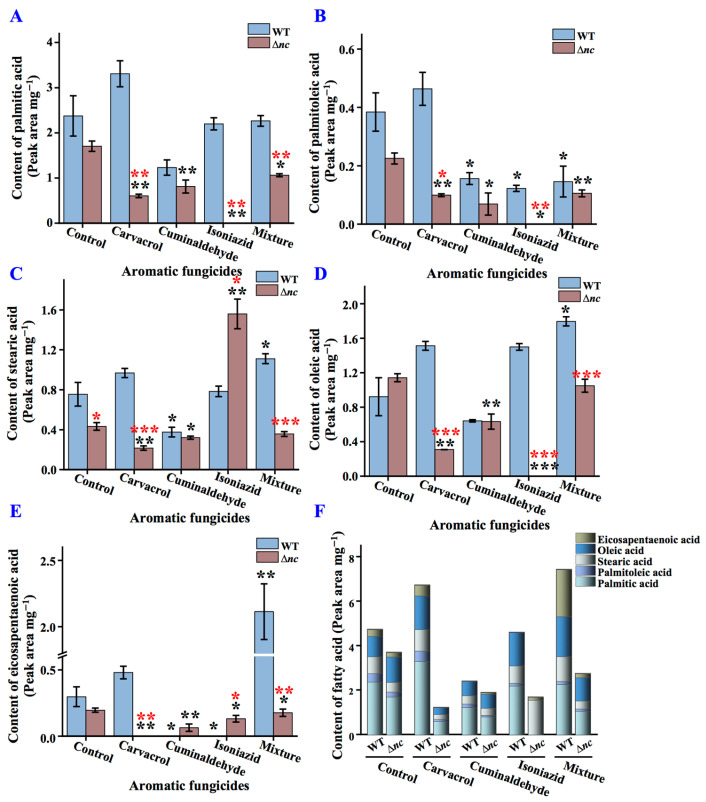
The impact of aromatic fungicides on fatty acid content in wild-type and Δ*nc* strains. (**A**) The content of palmitic acid. (**B**) The content of palmitoleic acid. (**C**) The content of stearic acid. (**D**) The content of oleic acid. (**E**) The content of eicosapentaenoic acid. (**F**) The content of total fatty acid. WT, the wild-type strain. The mixture of the three compounds was prepared by weighing out carvacrol, cuminaldehyde and isoniazid in a ratio of 1:10:50. Values are means ± SD (*n* indicates triplicates), and error bars represent SD. Black asterisks indicate comparisons of the same strain under aromatic fungicide treatment and normal culture conditions; red asterisks indicate comparisons of Δ*nc* and wild-type strains under the same culture conditions. * *p* < 0.05, ** *p* < 0.01, and *** *p* < 0.001.

The fatty acid content and composition of the wild-type and Δ*nc* strains was determined by GC. As shown in Figure 10, under normal culture condition, compared with the wild-type strain, the content of palmitic acid, palmitoleic acid, stearic acid, and eicosapentaenoic acid in Δ*nc* decreased, resulting in 28.22% decrease in total fatty acid accumulation, but the content of oleic acid increased by 23.99%. Under carvacrol treatment, the wild-type strain showed elevated levels of palmitic acid, palmitoleic acid, stearic acid, oleic acid, and eicosapentaenoic acid, with 42.25% increase in total fatty acid content. In contrast, Δ*nc* exhibited decreased levels of all fatty acids, leading to 66.87% reduction in total fatty acid accumulation. Upon cuminaldehyde treatment, the fatty acid content of the wild-type and Δ*nc* strains decreased by approximately 49.16%. With isoniazid treatment, the total fatty acid content of the wild-type strain showed no significant difference, while the total fatty acid content of Δ*nc* decreased by 54.31%. Under mixture treatment, the total fatty acid content of the wild-type strain increased by 57.02%, with an increase in stearic acid, oleic acid, and eicosapentaenoic acid, but the total fatty acid content of Δ*nc* decreased by 25.69% (Figure 10).

## 4. Discussion

nCDase is a hydrolase of ceramide that has been implicated in multiple biologic processes [31,32]. The findings of this study elucidate the role of nCDase in *N. crassa*’s response to aromatic fungicides such as carvacrol, cuminaldehyde, and isoniazid. By integrating phenotypic and metabolism analyses, we demonstrate that nCDase-mediated cellular composition remodeling is central to aromatic fungicides’ stress adaptation, membrane integrity, and survival.

It is evidenced that the sphingolipid metabolism is a critical regulator of cellular responses to environmental stressors in eukaryotes [33,34]. Plants lyse sphingolipids on the cell membranes of oomycetes through ceramidase to enhance the defense response against microbial pathogens [35]. nCDase is an essential factor that controls intestinal immune cell dynamics in mice. Cellular nCDase or extracellular vesicle-related nCDase generates sphingosine, which promotes macrophage-driven Th1 immunity [36]. Song [11] found that novel bis-pyrazole carboxamide derivatives block multiple metabolic pathways by down-regulating the CAT and up-regulating ceramidase expression, thereby disrupting the structure and function of cell membrane, affecting cellular differentiation, and eventually leading to cell death. Our data revealed that nCDase promoted the synthesis of fatty acids and carotenoids in *N. crassa* and was associated with antioxidant enzyme activities, along with the composition and integrity of cell walls and cell membranes. It is speculated that nCDase may be one of the targets for inhibiting the growth of pathogenic fungi.

nCDase plays a role in resisting stress from fungicide agents. Carvacrol treatment caused morphological deformation of A. flavus, which is commonly found in moldy grains, and the resulting increased electrolyte leakage indicates damage to the plasma membrane [37]. Transcriptome analysis revealed that differentially expressed genes were mainly associated with fatty acid degradation, autophagy, peroxisomes, the tricarboxylic acid cycle, oxidative phosphorylation, and DNA replication in *A. flavus* mycelia exposed to carvacrol [37]. Carvacrol stimulated chitin synthesis while suppressing carotenoid accumulation in wild-type and Δ*nc* strains. In addition, carvacrol stress enhanced fatty acid biosynthesis in the wild-type strain, yet inhibited fatty acid production and elevated CAT activity in Δ*nc*. This aligns with the observations in *Candida auris*, where the antioxidant enzymes’ activities induced by antifungal agents triggered oxidative membrane damage and cell death [38]. It was reported that cuminaldehyde inhibited the mycelial growth of *Penicillium italicum* that causes Penicillium disease in citrus fruits. In this study we found that cuminaldehyde suppressed conidiation and fatty acid biosynthesis in wild-type and Δ*nc* strains of *N. crassa*, while modifying cell wall and cell membrane composition and enhancing carotenoid production in Δ*nc*. Cuminaldehyde-induced oxidative stress triggered reactive oxygen species accumulation, compromised plasma membrane integrity and permeability, and intracellular content leakage [17]. Isoniazid stress exposure compromised the collapse and deformation of conidia and the leakage of nucleic acid in the wild-type and Δ*nc* strains, concomitant with reduced total carotenoids biosynthesis. In Δ*nc*, isoniazid inhibited fatty acid synthesis, induced chitin synthesis, but reduced ergosterol synthesis. Isoniazid exposure induces toxin-antitoxin gene expression in Mycobacterium tuberculosis [39]. The mixture of the three compounds resulted in mycelial breakage in the wild-type strain and conidial collapse in Δ*nc*. Mixture stress reduced ergosterol synthesis and carotenoid synthesis in the wild-type and Δ*nc* strains while leading to nucleic acid leakage. The inhibition efficiency of the mixture was higher, presumably due to the three compounds acting synergistically or the three compounds react to form new product with enhanced inhibitory activity. The synergistic effects of carvacrol, cuminaldehyde, and isoniazid highlight how combinatorial antifungal strategies amplify membrane destabilization. We will further study the mechanism by which the mixture inhibits the growth of fungi effectively, develop new fungicides, search for new targets, and reduce the harm caused by pathogenic fungi. In summary, common stress responses across the three compounds included increased chitin synthesis and membrane damage. Compound-specific effects were also observed that carvacrol enhanced fatty acid biosynthesis, cuminaldehyde increased carotenoids, and isoniazid reduced ergosterol synthesis. The Δ*nc* strain exhibited differential sensitivity to each compound, indicating that nCDase deficiency alters stress adaptation pathways in a compound-specific manner.

In addition, the synthesis of fatty acid starts with acetyl coenzyme A as the initial substrate, which undergoes condensation to form malonyl coenzyme A, and further extends the carbon chain [40]. The biosynthesis of ergosterol begins with acetyl coenzyme A and is catalyzed by enzymes such as squalene epoxidase and squalene synthase [41]. Carotenoids are mainly synthesized through the mevalonate pathway. Acetyl coenzyme A is the substance for the synthesis of isoprene units, which are further polymerized to synthesize carotenoids [42]. All the precursors of fatty acid, carotenoid and ergosterol synthesis is acetyl coenzyme A. The proportion of fatty acids affects membrane stability, thereby non-specifically influencing the entry of fungicides [43]. Ergosterol is the target of fungicides. Its content also affects the fluidity, stability and permeability of the cell membrane [44,45]. Carotenoids are potent antioxidants that quench ROS and protect membrane lipids from oxidative damage. Ceramidase catalyzes the hydrolysis of ceramide to sphingosine and fatty acid [46]. The core structure of ceramides is sphingosine linked to long-chain fatty acids by amide bonds. The synthesis of these long-chain fatty acids is dependent on a fatty acid elongase system that shares acetyl coenzyme A and NADPH with conventional fatty acid synthesis [42]. Since the synthesis of fatty acids is crucial for cell membrane, in Δ*nc*, the increase in ergosterol synthesis compensated for the decrease in fatty acid content, thereby enhancing the stability of the cell membrane. Additionally, ceramide may be involved in stress signaling and indirectly affect carotenoid synthesis. Carotenoid is the general term for a class of natural pigments with coloring ability, and is a subfamily of terpenoids. The existence of conjugated double-bond system endowed carotenoid molecules with good antioxidant activity. *N. crassa* responds to the stress of aromatic fungicide agents by regulating the synthesis of fatty acids and carotenoids. The competition for acetyl-CoA—a shared precursor for fatty acid, ergosterol and carotenoid synthesis—emerges as a key metabolic node under fungicide stress. In the wild-type strain, carvacrol induced fatty acid synthesis to repair membrane damage, while Δ*nc* exhibited diminished fatty acid production and redirected resources toward carotenoid precursors. This metabolic trade-off highlights nCDase’s role in prioritizing lipid repair over antioxidant defense. The deletion of *nc* reduced neurosporaxanthin (a terminal carotenoid with potent antioxidant properties) may render it vulnerable to oxidative stress, despite compensatory increases in precursor carotenoids. Notably, the wild-type strain’s ability to maintain higher T-SOD and CAT activities under carvacrol stress suggests nCDase coordinates both lipid metabolism and redox homeostasis, possibly via sphingosine-mediated signaling. Upon stress, *N. crassa* regulated the permeability and stability of cell membranes by altering the composition of fatty acids and scavenging stress-generated reactive oxygen species by synthesizing carotenoids. nCDase reduced the damage caused by stress through altering the content and composition of fatty acids and carotenoids in *N. crassa*. However, it remains formally possible that the observed alterations in fatty acids and carotenoids represent a secondary adaptive response to cell membrane stress rather than a direct metabolic consequence of ceramide homeostasis disruption. Regardless of the mechanistic hierarchy, our data clearly establish a functional link between nCDase activity and lipid homeostasis under fungicide-challenged conditions.

This study provides evidence that nCDase-mediated metabolism is a central regulator of *N. crassa*’s adaptive responses to aromatic antifungal agents. Our findings highlight the role of nCDase in maintaining membrane homeostasis and orchestrating metabolic reprogramming under aromatic fungicides stress. Although *N. crassa* serves as a robust model for studying sphingolipid metabolism, the specific phenotypes reported here, particularly the compound-specific sensitivities and metabolic trade-offs, require direct validation in plant-pathogenic fungi before drawing generalizable conclusions about antifungal targeting. However, our results provide novel insights into fungal stress adaptation mechanisms and potential antifungal targets.

## Data Availability

Dataset available on request from the authors.

## Abbreviations

The following abbreviations are used in this manuscript:

NC	Neutral ceramidase (nCDase)
T-SOD	Total superoxide dismutase
CAT	Catalase
PI	Propidium iodide
